# Omani propolis: chemical profiling, antibacterial activity and new propolis plant sources

**DOI:** 10.1186/1752-153X-7-158

**Published:** 2013-09-22

**Authors:** Milena Popova, Rosa Dimitrova, Hassan Talib Al-Lawati, Iva Tsvetkova, Hristo Najdenski, Vassya Bankova

**Affiliations:** 1 Institute of Organic Chemistry with Centre of Phytochemistry, Bulgarian Academy of Sciences, Sofia 1113, Bulgaria; 2 Honey Bee Research Lab, Directorate General of Agriculture and Livestock Research, Ministry of Agriculture and Fisheries, Muscat, Sultanate of Oman; 3 Institute of Microbiology “Stefan Angelov”, Bulgarian Academy of Sciences, Sofia 1113, Bulgaria

**Keywords:** Propolis, Chemical profiling, Propolis plant sources, *Azadiracta indica*, *Acacia*, Antibacterial activity

## Abstract

**Background:**

Propolis (bee glue) is a resinous honeybee product having a long history of application in many countries as a traditional remedy for treating wounds, burns, soar throat, stomach disorders, etc. It has been proved to possess beneficial biological effects, including antimicrobial, antioxidant, anti-inflammatory, cytotoxic, antiulcer, and many others. Bees gather propolis from diverse resinous plant parts and in different phytogeographic regions its chemical composition might vary significantly. In this article we report the results of the first study on the chemical profiles of propolis from Oman, its plant origin and antibacterial activity.

**Results:**

The chemical profiles of Omani propolis extracts were obtained by GC-MS analysis after silylation. Over 50 individual compounds were identified in the samples, belonging to different compound types: sugars, polyols, hydroxy acids, fatty acids, cardanols and cardols, anacardic acids, flavan derivatives, triterpenes, prenylated flavanones and chalcones. The profiles were dissimilar from other known propolis types. They demonstrate that although Oman is not a large country, the plant sources of propolis vary significantly, even in the same apiary and the same season. Based on chemical profiles, and isolation and identification of major marker compounds (new propolis constituents), new plant sources of propolis were found: *Azadiracta indica* (neem tree) and *Acacia* spp. (most probably *A. nilotica*). The ethanol extracts of the studied propolis samples demonstrated activity against *S. aureus* (MIC < 100 μg. mL^-1^) and *E. coli* (MIC < 380 μg. mL^-1^).

**Conclusion:**

Omani propolis is different form the known propolis types and demonstrates significant chemical diversity. Its most important plant source is the resin of *Azadirachta indica*, and as a result its typical components are С_5_-prenyl flavanones. Other plant sources have been identified, too, playing some role in resin collection by bees in Oman: *Acacia* spp. (most probably *A. nilotica)* and *Mangifera indica.* The results demonstrate also the potential of Omani propolis as antimicrobial.

## Background

Propolis (bee glue) is a resinous honeybee product collected by bees from plants and used as a building material and as a defensive antimicrobial substance in their hives [1]. It has a long history of application in many countries as a traditional remedy for treating wounds, burns, soar throat, stomach disorders, etc [2]. In the last decades, the medicinal properties of propolis attracted the attention of scientists and nowadays a lot of data exist concerning its chemical composition and diverse biological effects, including antimicrobial, antioxidant, immunomodulating, anti-inflammatory, cytotoxic, antiulcer, and many other activities [2-5]. It is a popular ingredient of food supplements, health foods and beverages, cosmetics [6].

Bees gather propolis from diverse resinous parts of living plants and in different phytogeographic regions its chemical composition might vary significantly due to the specificity of the local flora and the choices it offers to bees. At present, several propolis types are known, according to their chemical profiles and source plants: poplar type (European) propolis, Brazilian green propolis (*Baccharis* type*)*, red South American propolis (*Dalbergia* type), Mediterranean propolis (rich in diterpenes from *Cupressus* spp.) [2], etc. Numerous studies have revealed that in different environments honeybees are capable of finding appropriate propolis floral sources with significant antimicrobial activities [7]. Because of this, new propolis types from unexplored regions of the world have the potential to provide valuable leads to secondary metabolites with important bioactivities. In this article we report the results of the first study on the chemical profiles of propolis from Oman, its plant origin and antibacterial activity.

## Results and discussion

### Chemical profiles

**Figure 1:**
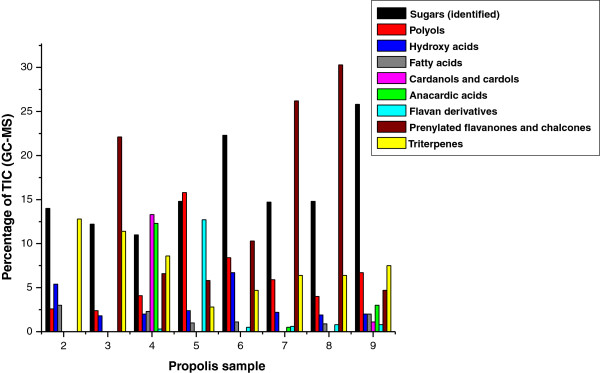
Chemical profiles of Omani propolis extracts.

The chemical profiling of propolis extracts was performed by GC-MS analysis. Over 50 individual compounds were identified in the samples (see Additional file 1: Table S1), belonging to different compound types: sugars, polyols, hydroxy acids, fatty acids, cardanols and cardols, anacardic acids, flavan derivatives, triterpenes, prenylated flavanones and chalcones. A representation of the chemical profiles by groups of compounds is shown in Figure 1. It is obvious that the sample profiles display significant chemical differences, both qualitative and quantitative. The profiles demonstrate that although Oman is not a large country, the plant sources of propolis vary significantly, even in the same apiary and the same season. In addition, they are completely dissimilar from poplar type (European) propolis, and from the recently described Mediterranean propolis. Omani samples do not contain phenolic acids and their esters found in poplar propolis [8]. The typical poplar flavonoids pinocembrin, pinobanksin 3-O-acetate, galangin, and chrysin [9] are not present, either. Further, the samples do not contain any diterpenes, which are characteristic for Mediterranean type propolis [10].

**Figure 2:**
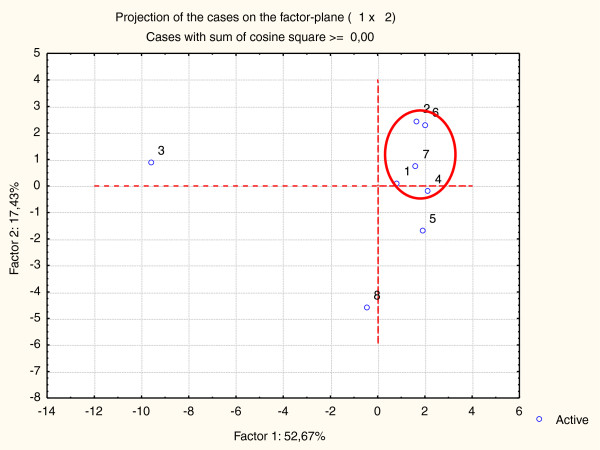
PCA of propolis chemical profiles of Omani propolis samples.

In order to analyze the large amount of analytical data we applied chemometric approach: the Principal Component Analysis (PCA). The central idea of PCA is to reduce the dimensionality of a data set in which there are a large number of correlated variables, while retaining as much as possible the total information. We used for PCA analysis the relative amounts of individual constituents of propolis from the GC-MS analysis (data from Additional file 1: Table S1). The obtained two-dimensional plot (Figure 2) covers 78% of the total variation. One well defined group is formed by samples OM-1, OM-2, OM-4, OM-6 and OM-7. These samples (excluding OM-1) are characterized by relatively high concentration of prenylated flavanones and chalcones.

Concerning the presence of chalcones, it is important to note that flavanones can easily turn to chalcones under the conditions of silylation for GC-MS analysis [11]. In order to check whether the chalcones are native constituents of Omani propolis, we examined the ^1^H-NMR spectra of the extracts from the latter samples, looking for the typical doublets (J ~ 16 Hz) of the α- and β-protons of the chalcone skeleton in the range δ 6.7 – 7.4 and δ 7.3 – 8.0 [12]. We were unable to detect any such signals in the ^1^H-NMR spectra, which lead to the conclusion that chalcones registered in GC-MS are artifacts produced from the corresponding flavanones and are not present in the native propolis.

**Figure 3:**
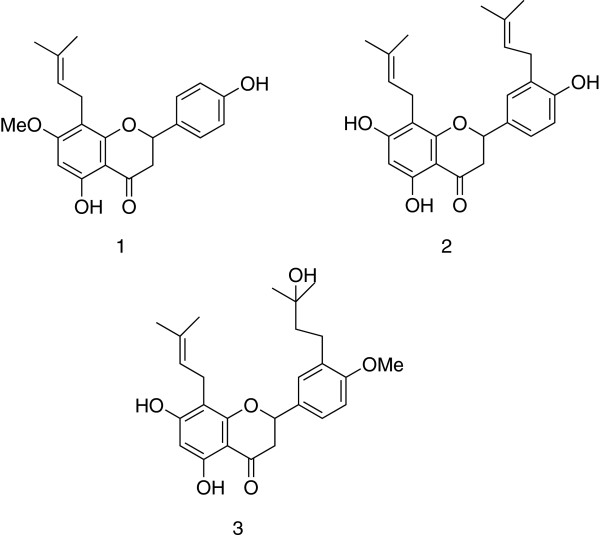
**Omani propolis constituents originating from *Azadiracta indica.***

Till now, several prenylated flavanones (propolins) have been found in propolis from Pacific islands: Taiwan [13], Okinawa [14], Indonesia [15]; the plant sources of these compounds were identified as *Macaranga* species. All of them contain C10 prenyl moieties in their molecules, while the flavanones identified in Omani samples contained C5 prenyl side chains. Obviously, the floral source of the latter flavanones has to be some plant different from *Macaranga*. In order to identify this floral source, the complete structure elucidation of the major prenylated flavanones was necessary. Mass spectral data of silylated flavanones (See Additional file 1: Table S2) allow the identification of the type and number of substituents (OH-, OMe-, prenyl- groups) and their location in rings A or B of the flavanone frame. The exact position of the prenyl groups, however, cannot be detected from the mass spectrum. For this reason, isolation of important constituents of sample OM-6 was performed. Its ethanol extract afforded, after repeated chromatographic separations, two pure individual compounds. By comparison of their spectral properties (NMR spectra, MS) with literature data [16], these compounds were identified as 7-O-methyl-8-prenylnaringenin **1** and 3’,8-diprenylnaringenin **2** (Figure 3). Both compounds are new propolis constituents and have been isolated previously from the neem tree *Azadiracta indica*[16,17]. For this reason we consider this tree as a likely major plant source of the samples OM-2, OM-6 and OM-7.

The adaptation of *A. indica* to hot and dry climates has made it one of the most commonly planted species in arid and semi-arid areas [18], and it is widespread in Oman [19]. The material collected by bees seems to be the resin, produced by resin glands on the leafy shoots of neem trees [20]. The chemical composition of this resin is largely unknown; only one prenylated flavanone derivative, 8-prenyl-5,7-dihydroxy-3’-(3-hydroxy-3-methylbutyl)-4’-methoxyflavanone **3** has been identified in it so far. This flavanone has been isolated from neem resin glands as a new natural product [21] and no other source of **3** has yet been reported, thus it is a good taxonomic marker for *A. indica* resin. Compound **3** was detected in samples OM-2, OM-6 and OM-7. It was tentatively identified in the GC-MS profiles of the samples on the basis of its characteristic mass spectral fragmentation. The spectrum of the silylated compound is characterized by a low intensity molecular ion peak at *m/z* 656, corresponding to a molecular formula C_26_H_32_O_6_ and base peak at *m/z* 641 [M-15]^+^, This molecular formula corresponds to a flavanone with one prenyl group, one hydroxylated pentyl group, two OH and one OCH_3_ groups. The mass spectrum displays the typical fragments of a flavanone bearing 5 and 7 hydroxyl groups and one prenyl group in ring A: ions at *m/z* 349 [A_1_ – CH_3_]^+^, 321 [A_1_ – CH_3_ - CO]^+^, 309 (98%) [A_1_ – C_4_H_7_]^+^[22]. On the other hand, the presence of a non-aromatic silyloxy group is indicated by the ion *m/z* 552 [M-CH_3_-OTMS]^+^. The position of the methyl group and the hydroxylated pentyl group in ring B are supported by the presence of the fragment ions at *m/z* 293 [B_2_]^+^ and 291 [B_3_]^+^. The peak at *m/z* 497, resulting from the loss of the hydroxylated side chain, supports the structure 8-prenyl-5,7-dihydroxy-3’-(3-hydroxy-3-methylbutyl)-4’-methoxyflavanone **3** (analogous peak was detected in the spectrum of the unsilylated compound [21]). The occurrence of compound **3** in Omani propolis thus provides an evidence for the contribution of *A. indica* as propolis source.

This finding adds a new species to the list of propolis source plants. It is important to note that *A. indica* is one of the most widely used medicinal plants in India since ancient times [23]; it possesses diverse biological activities, including antibacterial, antiviral and antifungal properties. Antifungal activity has been demonstrated for some individual neem constituents, including compound **3**[23]. Considering this fact, the choice of bees for resin source is not surprising.

The prenylated flavanones were detected in all Omani samples studied excluding OM-1. In the latter sample, the only secondary metabolites found were triterpenic compounds (individual compounds could not be identified), their source remaining unclear.

**Figure 4:**
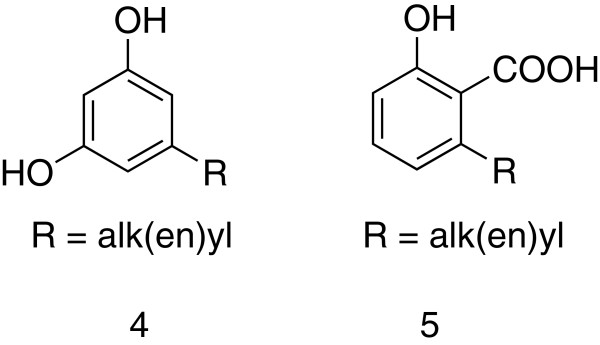
**Omani propolis constituents originating from *Mangifera indica.***

Sample OM-3 has a profile different form the ones on the other samples, its major constituents being cardanol (alkylphenol), cardols [alk(en)ylresorcinols)] **4** and anacardic acids **5** (Figure 4). These three related compound types have been found in propolis samples from Brazil [24], while cardols have been detected in poroplis from Thailand and Indonesia [15,25]. They most probably originate from *Mangifera indica* fruit bark and are known antifungal substances [15,26]. Sample OM-3 contained also significant amounts of C_5_-prenylated flavanones (and the resulting chalcones), as well as triterpenes. Obviously, this particular sample is of at least dual origin: *Mangifera indica* and *Azadiracta indica.* The contribution of *M. indica* as a resin source could be detected also in sample OM-8, which comes from the same region as sample OM-3 (Saham). In OM-8 however, these compounds are present in much lower concentration compared to OM-3. Nevertheless, this fact suggests that the bees in this region have access to both propolis plant sources.

**Figure 5:**
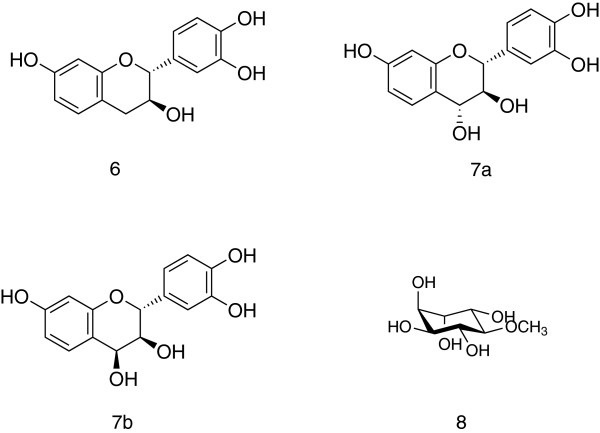
**Omani propolis constituents originating from *Acacia *spp.**

The sample OM-4 is unique among the studied samples in that it contains high amounts of flavan derivatives. The mass spectra of their TMS ethers were similar to those of catechine derivatives with two OH groups in ring B [27]. Catechins are known constituents of *Azadirachta indica*[28], however, the samples where the *Azadirachta* prenylflavanones are major components contained only minor amounts of these flavans. For this reason, their positive identification was necessary and several compounds were isolated from this propolis sample using consecutive chromatographic procedures. Two pure substances were obtained and characterized by mass and NMR spectra and comparison with literature data as fisetinidol **6**[27,29] (Figure 5) and a mixture of two stereoisomers of mollisacacidin: 2,3-*trans-*3,4-*trans* (**7a**) and 2,3-*trans-*3,4-*cis* (**7b**) mollisacacidin (Figure 5) [30,31]. All three compounds are new propolis constituents, and flavan-3,4-diols have not been found in propolis till now.

Another feature of this sample is the high concentration of the cyclitol derivative pinitol **8**, also new for propolis. Indeed, pinitol has been found in several honey types but always as a minor constituent [32], so it is highly improbable that it has come into propolis from honey, unlike glucose and other carbohydrates. Pinitol together with the flavan derivatives form a combination of phytochemicals that can point to the plant source of this particular propolis sample: it is a combination typical for many *Acacia* species [33,34]. Taking into consideration the distribution of *Acacia nilotica* in Oman [19], it is the most probable major source of OM-4. The presence of some amounts of prenylated flavanones/chalcones proves the importance of the neem tree as a secondary source for this sample.

It is noteworthy that all samples contain triterpenes but different samples contain different triterpenes and many of them could not be identified by GC-MS. The one common for all samples was cycloartenol. The exact plant origin of the triterpene compounds is yet unknown, but triterpenes are widely distributed in plant kingdom and the potential sources are numerous.

In this study, eight propolis samples were studied. Six of them were collected at the same apiary (region of Rumai) in different months of 2011, and only two samples (OM-3 and OM-8) originated from another location, Saham. Surprisingly, samples collected at virtually the same time of year from the same apiary from neighboring hives demonstrated different chemical profiles, for example OM-4 (September 10^th^) and OM-5 (September 11^th^). On the other hand, samples from different seasons were similar: OM-2 (June) and OM-6 (September). This difference indicates that propolis collection could be directed by some random factors.

The two samples from Saham (OM-3 and OM-8) have in common the presence of cardols and anacardic acids which sets them apart from the other samples, but they also display considerable quantitative differences between them.

### Antimicrobial activity

The alcohol extracts of the studied Omani propolis samples were tested for their activity against *Staphylococcus aureus, Escherichia coli* and *Candida albicans.* Somewhat surprisingly, no antifungal activity against *C. albicans* could be detected (MIC > 1000 μg. mL^-1^). The results of the antibacterial tests are presented in Table 1.

**Table 1:** Antimicrobial activity of Omani propolis samples (extracts with 70% ethanol)

**Sample**	**Minimum Inhibitory concentration (μg. mL**^**-1**^**)**	
	***S. aureus***	***E. coli***
OM-1	43	334
OM-2	90	356
OM-3	169	338
OM-4	86	344
OM-5	88	350
OM-6	42	169
OM-7	43	172
OM-8	90	356
BG^a^	125	>1000
Netilmicin	4	10

^a^ Bulgarian (poplar type) propolis extract.

Al samples demonstrated good antibacterial activity, taking into consideration that natural products which produce minimum inhibitory concentrations (MIC) in the range 100–1000 mg mL^-1^ in *in vitro* susceptibility tests can be classified as antimicrobials [35]. The MIC for all tested samples against *E. coli* were higher (lower activity) than against *S. aureus.* Most Omani samples were more active than a typical poplar type propolis sample from Bulgaria, both against *S. aureus* and *E. coli*.

There was a statistically significant correlation (r = 0,9646; p < 0.01) between the MIC against *S. aureus* and *E. coli*. The most active samples against both microorganisms were OM-6 and OM-7, the samples richest in prenylated flavonoids. Prenylated flavonoids are known to possess antimicrobial activity [36,37] and that may be the explanation of this observation.

## Conclusion

The results obtained in the present study lead to the conclusion that Omani propolis is different form the known propolis types and demonstrates significant chemical diversity. Its most important plant source turns out to be the resin of *Azadirachta indica*, and as a result its typical components are С_5_-prenyl flavanones. Other plant sources have been identified, too, playing some role in resin collection by bees: *Acacia* spp. (most probably *A. nilotica)* and *Mangifera indica.* Further studies of propolis from the region of the Persian Gulf should clarify the importance of neem and *Acacia* species as propolis sources.

The results demonstrate also the potential of Omani propolis as antimicrobial. The presence of biologically active phenolic constituents (prenylflavanones, cardols, anacardic acids, etc.) is an indication for its potential for application in complementary and alternative medicine, cosmetics and health foods.

## Experimental

### Propolis samples

The samples were collected in 2011 (with one exception collected in 2012) at two locations. The exact time and site of collection are given in Table 2.

**Table 2:** Propolis samples

**Sample No**	**Origin**	**Balsam (%)**
OM-1	Rumais 24/5/11	36.4 ± 0.4
OM-2	Rumais 21/6/11	49 ± 1
OM-3	Saham 18/9/12	23.4 ± 0.7
OM-4	Rumais 10/9/11	34.8 ± 0.1
OM-5	Rumais 11/9/11	30 ± 1
OM-6	Rumais 6/9/11	35 ± 1
OM-7	Rumais 19/11/11	41 ± 2
OM-8	Saham - propolis on textile fabric	ND^a^

^a^ Not detrmined, propolis cannot be removed quantitatively from the fabric.

### Extraction and sample preparation

Propolis was cooled in a refrigerator, grinned and extracted twice with 70% ethanol (1:10, w:v) at room temperature for 24 h. A part of the ethanol extract (5 mL) was evaporated to dryness. The balsam yield was determined by two parallel measurements. (Balsam content could not be determind only in sample OM-9 because propolis couls not be separated quantitatively from the textile fabric). The dry extract (combined from the two parallel measurments) was further analyzed by GC-MS after silylation, as well as for its antimicrobial activity. About 5 mg of the extract were mixed with 50 μL of dry (water-free) pyridine and 75 μL of bis(trimethylsilyl)-trifluoroacetamide (BSTFA) and heated at 80°C for 20 min. The silylated extracts and reference compounds were analysed by GC– MS.

### GC-MS analysis

The GC–MS analysis was performed with a Hewlett–Packard gas chromatograph 5890 series II Plus linked to a Hewlett–Packard 5972 mass spectrometer system equipped with a 30 m long, 0.25 mm i.d., and 0.5 μm film thickness HP5-MS capillary column. The temperature was programmed from 60 to 300°C at a rate of 5°C/min, and a 10 min hold at 300°C. Helium was used as a carrier gas at a flow rate of 0.8 mL/min. The split ratio was 1:10, the injector temperature 280°C, the interface temperature 300°C, and the ionization voltage 70 eV. Every extract was analyzed in duplicate.

### Identification and quantification of compounds

The identification of individual compounds was performed using computer searches on commercial libraries, comparison with spectra of authentic samples and literature data. If no reference spectra were available, identification was performed based on the mass-spectral fragmentation, in such cases for some compounds only tentative structures were proposed. Some constituents remained unidentified because of the lack of relevant references and information (none of them major constituent). The quantification of individual constituents is based on internal normalization. The percentage figures in the tables refer to percent of the Total Ion Current, TIC, and are semi-quantitative.

### Isolation of individual compounds

^1^H NMR (600 MHz) and ^13^C NMR (150 MHz), Bruker AV 600; spectra were taken in CDCl3 (deuterated chloroform) for compounds **1** and **2**, in CD_3_OD (deuterated methanol) for compounds **6**, **7a** + **7b**.

Individual compounds were isolated from the extract of sample OM-6 (7.1 g). The ethanol extract was concentrated *in vacuo* and extracted successively with petrol ether (3 x) and CHCl_3_ mg (3 x). The chloroform extract was evaporated to yield 2 g dry extract, which was subjected to column chromatography on a silica gel column (30 mm d. – 600 mm h; 150 g. silica gel) eluted with light petroleum — EtOAc (95:5 to 100% EtOAc) and 30 fractions were obtained. Fraction 4 yielded 2 mg of 7-O-methyl-8-prenylnaringenin **1**, and fractions 9 – 11 yielded 67 mg of 3’,8-diprenylnaringenin **2**.

Further individual compounds were isolated from the ethanol extract of sample OM-4 (3 g). The ethanol extract was concentrated *in vacuo* and extracted successively with petrol ether (3 x), CHCl_3_ mg (3 x) and EtOAc (3x). The petrol ether fraction contained mainly triterpenoids and was not further analyzed. The ethyl acetate extract was evaporated to dryness (200 mg) and subjected to preparative thin layer chromatography (PTLC) (silica gel 60 F254 glass plates Merck, 20×20 cm, 0.25 mm, mobile phaseCHCl_3_ – MeOH 8: 2; detection under UV light 254 and 366 nm), and two substances were isolated: 1.2 mg of fisetinidol **6** and 3 mg of a mixture of 2,3-*trans-*3,4-*trans* mollisacacidin **7a** and 2,3-*trans-*3,4-*cis* mollisacacidin **7b**.

^1^H NMR (600 MHz) and ^13^C NMR (150 MHz), Bruker AV 600; spectra were taken in CDCl3 (deuterated chloroform) for compounds **1** and **2**, in CD_3_OD (deuterated methanol) for compounds **6**, **7a** + **7b**.

### Antimicrobial tests

The MIC of propolis extracts were determined using broth microdilution method with test strains *Staphylococcus aureus* 209, *Escherichia coli* WF + and *Candida albicans* 562 (obtained from the Bulgarian Type Culture Collection, institute for State Control of Drugs, Sofia). Stock solution of propolis extracts was prepared, as follows: 4–5 mg (exact weight) dry extract were dissolved in 1 mL 70% ethanol. This stock solution was used for serial dilution in a 96-wells microtiter microplate from 400 – 500 μg/mL to 200–250, 100–125, 50–62.5, 25–31.25, 12.25 -15.62, 6.12 - 7.81 μg/mL. For the broth microdilution test, 50 μL of bacterial suspension in exponential phase of the growth was added to the wells of a sterile 96-well microtiter plate already containing 50 μL of twofold serially diluted propolis extracts in growth medium. Control wells were prepared with culture medium and bacterial suspension only. Three wells of the microtitre plate were used for each concentration of tested propolis extracts as well as for control sample. Incubation of the microplate was done for 24 h in the cultivation conditions described above. The MIC is the concentration in the last well in the row where no development of the microorganism is detected.

### Statistical analysis

Multivariate analysis of propolis chemical profiles was performed by principal component analysis (PCA), using the GC-MS data for the identified compounds expressed as a percentage of the Total Ion Current, respectively. Statistica Version 8.0 was used for the analyses.

## Competing interest

The authors declare that they have no competing interests.

## Authors’ contributions

MP participated in the chromatographic and the identification of individual compounds, RD performed the GC-MS analysis, HTAL collected the propolis samples and contributed to drafting the manuscript, IT and HN performed antimicrobial tests, VB conceived of the study, participated in its design and coordination and contributed to drafting the manuscript. All authors read and approved the final manuscript.

## Supplementary Material

Additional file 1: Table S1 Chemical profiles of Omani propolis ethanol extracts by GC-MS. **Table S2.** Important ions in the mass spectra of silylated compounds in Omani propolis samples (GC-MS). Click here for file
