# Supplementary material for: Omani propolis: chemical profiling, antibacterial activity and new propolis plant sources
Source: Chem Cent J. 2013 Sep 22;7:158. doi: 10.1186/1752-153X-7-158 (PMC3851436; doi:10.1186/1752-153X-7-158)
Supplement: Additional file 1: Table S1 — Chemical profiles of Omani propolis ethanol extracts by GC-MS. Table S2. Important ions in the mass spectra of silylated compounds in Omani propolis samples (GC-MS). [file 1752-153X-7-158-S1.doc]

Table S1. Chemical profiles of Omani propolis ethanol extracts by GC-MS

| **Compound** | **OM-1** | **OM-2** | **OM-3** | **OM-4** | **OM-5** | **OM-6** | **OM-7** | **OM-8** |
| --- | --- | --- | --- | --- | --- | --- | --- | --- |
| ***Sugars (identified)*** | **14.0** | **12.2** | **11.0** | **14.8** | **22.3** | **14.7** | **14.8** | **25.8** |
| β-fructofuranose | 3.2 | 2.2 | 2.2 | 5.0 | 5.2 | 3.2 | 2.5 | 8.0 |
| α-fructofuranose | 6.6 | 5.3 | 5.1 | 5.1 | 10.0 | 7.1 | 6.3 | 9.6 |
| α-glucopyranose | 1.4 | 1.4 | 1.3 | 1.7 | 2.8 | 0.1 | 1.9 | 3.5 |
| β-glucopyranos | 2.3 | 2.4 | 1.9 | 1.4 | 3.3 | 3.4 | 2.9 | 3.1 |
| Sucrose | 0.5 | 0.9 | 0.5 | 1.6 | 1.0 | 0.9 | 1.2 | 1.6 |
| ***Polyols*** | ***2.6*** | ***2.4*** | ***4.1*** | ***15.8*** | ***8.4*** | ***5.9*** | ***4.0*** | ***6.7*** |
| Glycerol | 1.4 | 0.7 | 2.0 | 1.3 | 3.0 | 0.9 | 0.8 | 2.8 |
| Erythritol | - | - | - | - | - | - | - | 1.2 |
| Mannitol | - | - | 0.2 | 0.3 | 0.5 | - | - | - |
| Inositol | 0 | 1.4 | 0.6 | 0.4 | 1.1 | 2.0 | - | - |
| Pinitol | 1.2 | 0.3 | 1.3 | 13.8 | 3.8 | 3.0 | 3.2 | 2.7 |
| ***Hydroxy acids*** | ***5.4*** | ***1.8*** | ***2.0*** | ***2.4*** | ***6.7*** | ***2.2*** | ***1.9*** | ***2.0*** |
| Mallic acid | 0.4 | 0.6 | 0.2 | 1.6 | 1.2 | 0.8 | 0.5 | 0.4 |
| Gluconic acid | 5.0 | 1.2 | 1.8 | 0.8 | 4.5 | 1.4 | 1.4 | 1.6 |
| ***Fatty acids*** | ***3.0*** | ***-*** | ***2.3*** | ***1.0*** | ***1.1*** | ***-*** | ***0.9*** | ***2.0*** |
| Palmitic acid | 0.9 | - | 0.7 | 0.5 | 0.3 | - | 0.4 | 0.8 |
| Stearic acid | 2.1 | - | 1.6 | 0.5 | 0.8 | - | 0.5 | 1.2 |
| ***Alkylpheniols and alkylresorcinols*** | ***0*** | ***0*** | ***13.3*** | ***0*** | ***0*** | ***0*** | ***0*** | ***1.1*** |
| Alkylphenol C15 | - | - | 0.2 | - | - | - | - | - |
| Alkyl resorcinol C15:1 | - | - | 0.4 | - | - | - | - | - |
| Alkyl resorcinol C15 | - | - | 1.1 | - | - | - | - | 0.2 |
| Alkyl resorcinol C17:2 | - | - | 2.4 | - | - | - | - | 0.3 |
| Alkyl resorcinol C17:3 | - | - | 0.3 | - | - | - | - | 0.1 |
| Alkyl resorcinol C17:1 | - | - | 0.8 | - | - | - | - | 0.3 |
| Alkyl resorcinol C17:1 | - | - | 3.2 | - | - | - | - | 0.1 |
| Alkyl resorcinol C17 | - | - | 1.3 | - | - | - | - | 0 |
| Alkylresorcinol C19:1 | - | - | 3.6 | - | - | - | - | 0.1 |
| ***Anacardic acids*** | ***-*** | ***-*** | ***12.3*** | ***-*** | ***-*** | ***-*** | ***-*** | ***3.0*** |
| Anacardic acid C15 | - | - | 0.9 | - | - | - | - | 0.6 |
| Anacardic acid C17:2 | - | - | 1.3 | - | - | - | - | 0.7 |
| Anacardic acid C17:1 | - | - | 1.0 | - | - | - | - | 0.2 |
| Anacardic acid C17:1 | - | - | 2.6 | - | - | - | - | 0.4 |
| Anacardic acid C17 | - | - | 1.6 | - | - | - | - | 0.5 |
| Anacardic acid C19:1 | - | - | 4.9 | - | - | - | - | 0.6 |
| ***Flavan derivatives*** | ***-*** | ***-*** | ***0.3*** | ***12.7*** | ***-*** | ***0.6*** | ***0.8*** | ***0.8*** |
| Fisetinidol | - | - | 0.3 | 2.5 | - | 0.6 | 0.8 | 0.8 |
| Mollisacacidin | - | - | - | 7.3 | - | - | - | - |
| Mollisacacidin (stereoisomer) | - | - | - | 2.9 | - | - | - | - |
| ***Prenylated flavanones and chalcones*** | ***-*** | ***22.8*** | ***6.6*** | ***5.8*** | ***10.7*** | ***26.6*** | ***30.9*** | ***4.7*** |
| Dihydroxy-methoxy-prenyl flavanone | - | 1.4 | - | - | - | - | - | - |
| Trihydroxy-methoxy-prenyl chalcone | - | 2.5 | 1.5 | 0.6 | 1.1 | 2.9 | 3.0 | 0.6 |
| Dihydroxy-methoxy-prenyl flavanone | - | - | - | 0.5 | 0.4 | 1.1 | 1.4 | - |
| Prenyl naringenin A | - | 0.7 | - | - | - | 0.6 | 0.9 | 0.4 |
| Trihydroxy-methoxy-prenyl chalcone isomer | - | 1.2 | 0.5 | - | 0.5 | 0.9 | 1.0 | 0.4 |
| Tetrahydroxy-prenyl chalcone | - | 2.9 | 0.9 | 0.3 | 0.5 | 1.4 | 1.6 | 1.1 |
| Prenylnaringenin B | - | 2.8 | - | 0.4 | - | 2.5 | 3.3 | - |
| Trihydroxy-methoxy-prenyl flavanone | - | - | - | - | - | 0.8 | 1.0 | - |
| Tetrahydroxy-prenyl chalcone (isomer) | - | 0.3 | - | - | 1.6 | - | 4.7 | - |
| Dihydroxy-methoxy-prenyl flavanone | - | 0.3 | - | 0.4 | 0.4 | - | - | - |
| Trihydroxy-methoxy-diprenyl chalcone | - | 2.6 | 1.8 | 0.8 | 2.0 | 4.0 | 3.9 | 0.8 |
| 8-prenyl-5.7-dihydroxy-3’-(3-hydroxy-3.3-dimethylbutyl)-4’-methoxyflavanone | - | 0.7 | - | - | 0.4 | 0.4 | 0.6 | - |
| Dihydroxy-methoxy-diprenyl flavanone | - | - | - | 1.6 | 0.9 | 3.0 | 2.0 | - |
| Tetrahydroxy diprenyl chalcone | - | 4.1 | 1.9 | - | 0.9 | 4.0 | 3.6 | 1.4 |
| Trihydroxi-diprenyl flavanone | - | 2.6 | - | 1.2 | 1.7 | 2.6 | 3.1 | - |
| Tetrahydroxy diprenyl chalcone | - | 0.7 | - | - | 0.3 | 2.4 | 0.8 | - |
| ***Triterpenes*** | ***12.8*** | ***11.4*** | ***8.6*** | ***2.8*** | ***4.7*** | ***6.4*** | ***6.4*** | ***7.5*** |
| Amyrin | - | - | 2.5 | - | 0.5 | - | 0.8 | 1.2 |
| Triterpenic alcohol amyrin type | - | - | - | - | 0.5 | - | - | - |
| Cycloartenol | 2.1 | 5.4 | 4.9 | 1.4 | 1.7 | 4.3 | 3.7 | 1.6 |
| Triterpene | - | 3.4 | - | - | 0.4 | 1.1 | - | 0.5 |
| Triterpene | 10.7 | - | - | - | - | - | - | - |
| Triterpenic ketone | - | - | - | 1.4 | - | - | - | - |
| Triterpene | - | 2.6 | - | - | 1.0 | 1.0 | 1.9 | 1.9 |
| Triterpene | - | - | 1.2 | - | 0.6 | - | - | - |
| Triterpenic acid | - | - | - | - | - | - | - | 2.3 |

Table S2. Important ions in the mass spectra of silylated compounds in Omani propolis samples (GC-MS)

| **No** | **Compound** | **Ions [*m/z* (aboundance)]** |
| --- | --- | --- |
| **1** | 7-O-methyl-8-prenylnaringenin | 498, M^+^**.**,(10), 483 (100), 306 (10), 291 (32), 263 (30), 251 (41) |
| **2** | 3’,8-diprenylnaringenin | 624, M^+^**.** (20), 609 (100), 364 (15), 349 (42), 321 (27), 309 (48) |
| **3** | 8-prenyl-5,7-dihydroxy-3’-(3-hydroxy-3-methylbutyl)-4’-methoxyflavanone | 656, M^+^**.** (2), 641 (13), 364 (11), 349 (48), 321 (28), 309 (67) |
| **4** | Cardol (C17:0) | 429 M^+^**.** (32), 477 (6), 218 (13), 268 (100) |
| **5** | anacardic acid (C17:0) | 546 M^+^**.** (7), 531 (100), 456 ( 441, 219 |
| **6** | fisetinidol | 562, M^+^**.**(21), 547 (1), 368 (100), 267 (32), 179 (9) |
| **7** | mollisacacidin | 650, M^+^**.**(0.5), 635 (0.5), 368 (100), 355 (31), 355 (28), 267 (10) |
| **8** | pinitol | 554, M^+^**.**(1), 318 (85), 305 (58), 260 (100), 217 (85) |
